# Supplementary material for: Epidemiology and SARS-CoV-2 Infection Patterns among Youth Followed at a Large Los Angeles Health Network during 2020–2022: Clinical Presentation, Prevalent Strains, and Correlates of Disease Severity
Source: Vaccines (Basel). 2023 May 25;11(6):1024. doi: 10.3390/vaccines11061024 (PMC10300956; doi:10.3390/vaccines11061024)
Supplement: Supplementary file 1 [file vaccines-11-01024-s001.zip › vaccines-2378913-supplementary.pdf]

Table S1. Demographic characteristics and outcomes of MISC in Los Angeles County youth.

	March 13, 2020-March 31, 2021 (N=11)		April 1, 2021-March 31, 2022 (N=14)	
Sex	N	%	N	%
Female (N, %)	4	36%	7	50%
Male (N, %)	7	54%	7	50%
Age group at testing	N	%	N	%
< 1 year (N, %)	0	0%	1	7%
1 - 5 years (N, %)	1	9%	5	36%
6 -11 years (N, %)	6	55%	4	29%
12 – 18 years (N, %)	4	36%	4	29%
19 – 24 years (N, %)	0	0 %	0	0%
Mean Age (years)	12.5		6.4	
**Race/ethnicity**	**N**	%	N	%
Asian (N, %)	0	0%	1	7%
Black/African American (N, %)	1	9%	3	21%
White (N, %)	3	27%	7	50%
Hispanic (N, %)	6	55%	3	21%
Comorbidities	3	27%	1	7%
**Outcomes**	**N**	%	N	%
Death (N, %)	1	9%	1	7%
Survival (N, %)	10	91%	13	93%

Table S2. SARS-COV-2 reinfection by subgroup in year 2 (N=109).

	N (%)
**Sex**	
Female	50 (45.9%)
Male	59 (54.1%)
**Age group at testing**	
< 1 year	4 (3.7%)
1 - 5 years	20 (18.3%)
6 -11 years	26 (23.9%)
12 – 18 years	26 (23.9%)
19 – 24 years	33 (30.3%)
Mean Age (SD)	13.2 (7.5)
**Race/ethnicity***	
Asian	10 (9.2%)
Black/African American	11 (10.1%)
Hispanic/Latino	47 (43.1%)
White	48 (44%)
Other	0 (0%)
Missing	0 (0%)
**Type of Insurance**	
Uninsured	2 (1.8%)
Medical/Safety Net	27 (24.8%)
HMO/Private	80 (73.4%)
**Comorbidities**	
Obesity	18 (16.5%)
Pulmonary	25 (22.9%)
Cardiac	5 (4.6%)
Other	0 (0%)
None	66 (60.6%)
**Occupation (Patient)**	
Working	9 (8.3%)
Student	95 (87.2%)
Disabled	2 (1.8%)
Other	3 (2.8%)
**Disease Severity**	
Asymptomatic	30 (27.5%)
Mild	74 (67.9%)
Moderate	4 (3.7%)
Severe or Critical	1 (0.9%)

Data are shown for Year 2 because almost all reinfections occurred during this period.

*Individuals could self-identify as ≥ 1 race/ethnicity
